# Whole-Genome Sequencing and *De Novo* Assembly of 67 Staphylococcus pseudintermedius Strains Isolated from the Skin of Healthy Dogs

**DOI:** 10.1128/mra.00039-22

**Published:** 2022-03-02

**Authors:** Norma Fàbregas, Daniel Pérez, Joaquim Viñes, Rocío Fonticoba, Anna Cuscó, Lourdes Migura-García, Lluís Ferrer, Olga Francino

**Affiliations:** a Vetgenomics, Edifici EUREKA, PRUAB, Campus UAB, Barcelona, Spain; b Department of Animal Medicine and Surgery, Universitat Autònoma de Barcelona, Barcelona, Spain; c IRTA, Centre de Recerca en Sanitat Animal (CReSA, IRTA-UAB), Barcelona, Spain; d SVGM, Molecular Genetics Veterinary Service, Universitat Autònoma de Barcelona, Barcelona, Spain; University of Rochester School of Medicine and Dentistry

## Abstract

We have *de novo* assembled 67 Staphylococcus pseudintermedius genomes, with median values of 2.6 Mbp size and 99.43% completeness, 2,386 coding sequences, 19 complete rRNAs, 59 tRNAs, and 4 noncoding RNAs. We released 51 single-contig complete genomes and 16 genomes with a circular main contig using Nanopore sequencing.

## ANNOUNCEMENT

Staphylococcus pseudintermedius is a commensal of the skin of dogs and the main causative agent of canine pyoderma (1), but populations inhabiting the skin of healthy dogs are largely unknown. The current paradigm indicates that infection arises when the skin barrier is altered by predisposing factors (2). We previously demonstrated that Nanopore sequencing allows *de novo* assembly of the entire genome of S. pseudintermedius (3). Here, we retrieved 67 S. pseudintermedius genomes from isolates from the skin of healthy dogs using long-read Nanopore sequencing.

Samples were obtained by rubbing sterile swabs on four skin sites from nine healthy dogs—perinasal, perioral, inguinal, and perianal. After culture in blood agar at 37°C for 24 h, colonies grown with the morphology of S. pseudintermedius (small silver colonies) were seeded in 3 mL of brain heart infusion (BHI) at 37°C for 16 h.

DNA was extracted with a ZymoBIOMICS DNA miniprep kit (Zymo Research). DNA quality and quantity were determined using a NanoDrop 2000 instrument and a Qubit double-stranded DNA (dsDNA) broad range (BR) assay kit (Fisher Scientific). The sequencing libraries were prepared with the rapid barcoding sequencing kit (SQK-RBK004; Oxford Nanopore Technologies [ONT]). Up to 12 barcoded samples were loaded in a MinION FLO-MIN106 9.4.1 flow cell and sequenced in a MinION Mk1B or Mk1C device (ONT). The fast5 files were base-called and demultiplexed, and adapters were trimmed with Guppy 5.0.11 (4) (ONT) (--dna_r9.4.1_450bps_sup.cfg) (--config configuration.cfg --barcode_kits SQK-RBK004 --trim_barcodes; min_score threshold default 60). Reads with a quality score lower than 10 were discarded. Run summary statistics were obtained with Nanoplot 1.38.1 (5) (--N50 --fastq).

Isolates were confirmed as S. pseudintermedius by EPI2ME WIMP workflow (6). Genomes were *de novo* assembled using Flye 2.8.3 (7) (--nano-raw --plasmids --trestle), except HSP279 and HSP281, which were assembled with Flye 2.9 (--nano-hq). Contigs were polished with medaka 1.4.3 (8) (medaka_consensus; -m r941_min_sup_g507). Genome completeness and contamination were assessed with CheckM 1.1.3 (lineage_wf) (9). Circlator 1.5.5 was used to rotate the genomes, fixing the start with the *dnaA* gene (10) (fixstart --min_id 70). Genomes were annotated with the NCBI Prokaryotic Genome Annotation Pipeline 5.2 and 5.3 (11). Multilocus sequence types (MLSTs) were assigned with PubMLST (https://pubmlst.org/; accessed November 2021) (12).

Nanopore sequencing allowed successful *de novo* assembly and polishing of 67 S. pseudintermedius genomes (Table 1). The average read *N*_50_ value was 5,270.77 bp with 133,226.86 reads per sample. The median values were 154× coverage (33 to 398×), 99.43% completeness (96.9 to 99.4%) as in previous hybrid assemblies (13), 2.6 Mbp for contig *N*_50_ value and genome size (2.5 to 2.9 Mbp), 37.6% GC content (37.2 to 37.8%), 2,386 coding DNA sequences (CDS) (2,247 to 2,805), 19 complete rRNAs (6 to 8 5S, 5 to 7 26S, and 5 to 8 23S), 59 tRNAs (58 to 61), and 4 noncoding RNAs (ncRNAs).

**TABLE 1:** Characteristics and accession numbers of high-quality genome assemblies from 67 Staphylococcus pseudintermedius isolates from 4 different skin sites of 9 healthy dogs^*a*^

ID	MLST	Source	Yr of isolation	Country of isolation	Assembly ID (accession version no.)	Assembly level	Genome accession no.	Genome assembly	No. of contigs	*N*_50_ contigs (bp)	Genome size of all contigs (bp)	No. of CDS (total)	Cov (×)	Comp (%)	Cont (%)	GC content (%)
HSP147	45^*b*^	Cf, I, H	2020	Spain	GCA_019997105.1	Compl	CP083230	Circular	1	2,596,738	2,596,738	2,379	199	97.96	0.57	37.6
HSP149	45^*b*^	Cf, I, H	2020	Spain	GCA_019997905.1	Compl	CP083229	Circular^*c*^	1	2,592,238	2,592,238	2,384	90	97.82	0.57	37.6
HSP151	45^*b*^	Cf, Pa, H	2020	Spain	GCA_019997185.1	Compl	CP083231	Circular^*c*^	1	2,596,528	2,596,528	2,380	134	97.96	0.57	37.6
HSP152	45^*b*^	Cf, Pa, H	2020	Spain	GCA_019997145.1	Compl	CP083228	Circular	1	2,596,499	2,596,499	2,386	78	96.92	0.57	37.6
HSP153	402^*b*^	Cf, Pa, H	2020	Spain	GCA_019997125.1	Compl	CP083227	Circular^*c*^	1	2,596,563	2,596,563	2,387	113	97.68	0.57	37.6
HSP154	45^*b*^	Cf, Pa, H	2020	Spain	GCA_019997265.1	Compl	CP083226	Circular	1	2,596,624	2,596,624	2,376	53	97.96	0.57	37.6
HSP155	45^*b*^	Cf, Pa, H	2020	Spain	GCA_019998705.1	Compl	CP083225	Circular	1	2,596,628	2,596,628	2,371	158	98.77	0.57	37.6
HSP156	45^*b*^	Cf, P, H	2020	Spain	GCA_019997565.1	Compl	CP083224	Circular^*c*^	1	2,596,695	2,596,695	2,374	132	98.11	0.57	37.6
HSP157	45	Cf, P, H	2020	Spain	GCA_019998065.1	Compl	CP083223	Circular^*c*^	1	2,596,646	2,596,646	2,381	151	98.25	0.57	37.6
HSP158	45^*b*^	Cf, P, H	2020	Spain	GCA_019997985.1	Compl	CP083222	Circular	1	2,596,630	2,596,630	2,391	120	97.85	0.57	37.6
HSP159	45^*b*^	Cf, P, H	2020	Spain	GCA_019998395.1	Compl	CP083221	Circular	1	2,596,316	2,596,316	2,377	90	97.66	0.57	37.6
HSP160	45^*b*^	Cf, P, H	2020	Spain	GCA_019998665.1	Compl	CP083220	Circular	1	2,596,481	2,596,481	2,388	38	97.57	0.57	37.6
HSP166	45^*b*^	Cf, Pn, H	2020	Spain	GCA_019997325.1	Compl	CP083219	Circular	1	2,596,789	2,596,789	2,375	148	97.82	0.57	37.6
HSP199	2189	Cf, Pa, H	2020	Spain	GCA_019998005.1	Compl	CP083218	Circular	1	2,574,155	2,574,155	2,380	33	99.29	0.00	37.6
HSP204	2190	Cf, Pa, H	2020	Spain	GCA_019997445.1	Compl	CP083217	Circular	1	2,470,602	2,470,602	2,260	33	98.30	0.00	37.8
HSP210	2191	Cf, P, H	2020	Spain	GCA_019998825.1	Compl	CP083216	Circular	1	2,472,661	2,472,661	2,249	191	99.43	0.00	37.8
HSP211	2175	Cf, P, H	2020	Spain	GCA_019998805.1	Compl	CP083215	Circular	1	2,472,653	2,472,653	2,249	136	99.43	0.00	37.8
HSP212	2175	Cf, P, H	2020	Spain	GCA_019998765.1	Compl	CP083214	Circular	1	2,472,754	2,472,754	2,257	158	99.43	0.00	37.8
HSP213	2175	Cf, P, H	2020	Spain	GCA_019998525.1	Compl	CP083213	Circular	1	2,472,662	2,472,662	2,247	398	99.43	0.00	37.8
HSP214	2192	Cf, Pn, H	2020	Spain	GCA_019998185.1	Compl	CP083212	Circular	1	2,524,384	2,524,384	2,319	119	99.43	0.00	37.8
HSP216	2176	Cf, Pn, H	2020	Spain	GCA_019998745.1	Compl	CP083211	Circular	1	2,611,897	2,611,897	2,403	97	99.43	0.57	37.6
HSP224	2177	Cf, P, H	2020	Spain	GCA_019998685.1	Compl	CP083210	Circular	1	2,711,397	2,711,397	2,501	88	99.43	0.57	37.6
HSP225	2177	Cf, P, H	2020	Spain	GCA_019998785.1	Compl	CP083209	Circular	1	2,752,898	2,752,898	2,571	289	99.43	0.57	37.6
HSP226	2177	Cf, P, H	2020	Spain	GCA_019997165.1	Compl	CP083208	Circular	1	2,750,631	2,750,631	2,567	227	99.43	0.57	37.6
HSP227	2177	Cf, P, H	2020	Spain	GCA_019996845.1	Compl	CP083207	Circular	1	2,750,563	2,750,563	2,568	212	99.43	0.57	37.6
HSP228	2177	Cf, P, H	2020	Spain	GCA_019997925.1	Compl	CP083206	Circular	1	2,750,560	2,750,560	2,568	195	99.43	0.57	37.6
HSP232	2178	Cf, Pa, H	2020	Spain	GCA_020685925.1	Contig	JAJEKF000000000	Circular	2	2,598,204	2,607,192	2,403	220	99.43	0.00	37.6
HSP235	551	Cf, Pn, H	2020	Spain	GCA_020685885.1	Contig	JAJEKT000000000	Circular	2	2,870,862	2,873,604	2,791	312	99.43	1.14	37.2
HSP236	551	Cf, Pn, H	2020	Spain	GCA_020685905.1	Contig	JAJEKS000000000	Circular	2	2,876,047	2,878,792	2,790	126	99.43	1.14	37.3
HSP237	551	Cf, Pn, H	2020	Spain	GCA_020685685.1	Contig	JAJEKR000000000	Circular	2	2,829,605	2,835,087	2,727	157	99.43	1.14	37.3
HSP238	45^*b*^	Cf, Pn, H	2020	Spain	GCA_019997285.1	Compl	CP083205	Circular	1	2,646,644	2,646,644	2,453	272	96.88	0.57	37.5
HSP239	45^*b*^	Cf, Pn, H	2020	Spain	GCA_019997805.1	Compl	CP083204	Circular	1	2,647,982	2,647,982	2,450	265	98.06	0.57	37.5
HSP240	551	Cf, P, H	2020	Spain	GCA_019998365.1	Compl	CP083203	Circular	1	2,870,858	2,870,858	2,760	169	99.43	1.14	37.2
HSP241	551	Cf, P, H	2020	Spain	GCA_020685845.1	Contig	JAJEKQ000000000	Circular	2	2,870,853	2,873,594	2,784	181	99.43	1.14	37.2
HSP242	551	Cf, P, H	2020	Spain	GCA_020685645.1	Contig	JAJEKP000000000	Circular	2	2,870,853	2,876,339	2,794	182	99.43	1.14	37.2
HSP243	551	Cf, P, H	2020	Spain	GCA_020685745.1	Contig	JAJEKO000000000	Circular	2	2,870,749	2,876,235	2,779	266	99.43	1.14	37.2
HSP244	551	Cf, P, H	2020	Spain	GCA_020685805.1	Contig	JAJEKN000000000	Circular	2	2,870,863	2,876,349	2,805	204	99.43	1.14	37.2
HSP245	2178	Cf, Pa, H	2020	Spain	GCA_020685785.1	Contig	JAJEKM000000000	Circular	3	2,598,196	2,609,478	2,399	198	99.43	0.00	37.6
HSP246	2178	Cf, Pa, H	2020	Spain	GCA_020685705.1	Contig	JAJEKL000000000	Circular	2	2,597,938	2,600,981	2,397	137	99.43	0.00	37.6
HSP249	2178	Cf, Pa, H	2020	Spain	GCA_020685725.1	Contig	JAJEKK000000000	Circular	2	2,597,934	2,600,977	2,396	148	99.43	0.00	37.6
HSP250	2178	Cf, I, H	2020	Spain	GCA_020685625.1	Contig	JAJEKJ000000000	Circular	2	2,598,182	2,601,232	2,405	175	99.43	0.00	37.6
HSP251	2178	Cf, I, H	2020	Spain	GCA_020685865.1	Contig	JAJEKI000000000	Circular	2	2,598,180	2,602,649	2,400	158	99.43	0.00	37.6
HSP252	2178	Cf, I, H	2020	Spain	GCA_019997885.1	Compl	CP083202	Circular	1	2,598,184	2,598,184	2,380	176	99.43	0.00	37.6
HSP253	2178	Cf, I, H	2020	Spain	GCA_020685765.1	Contig	JAJEKH000000000	Circular	2	2,598,192	2,604,278	2,398	206	99.43	0.00	37.6
HSP255	1026	Cf, Pn, H	2020	Spain	GCA_019997765.1	Compl	CP083201	Circular	1	2,599,010	2,599,010	2,377	131	98.86	0.00	37.6
HSP258	1026	Cf, P, H	2020	Spain	GCA_019997205.1	Compl	CP083200	Circular	1	2,599,012	2,599,012	2,370	120	98.30	0.00	37.6
HSP259	1026	Cf, P, H	2020	Spain	GCA_019997005.1	Compl	CP083199	Circular	1	2,599,031	2,599,031	2,383	155	98.86	0.00	37.6
HSP260	1026	Cf, P, H	2020	Spain	GCA_019997245.1	Compl	CP083198	Circular	1	2,599,024	2,599,024	2,375	71	99.24	0.00	37.6
HSP261	1026	Cf, P, H	2020	Spain	GCA_019997785.1	Compl	CP083197	Circular	1	2,599,041	2,599,041	2,372	145	99.43	0.00	37.6
HSP262	1026	Cf, P, H	2020	Spain	GCA_019997825.1	Compl	CP083196	Circular	1	2,599,036	2,599,036	2,382	147	99.43	0.00	37.6
HSP263	1026	Cf, I, H	2020	Spain	GCA_019997065.1	Compl	CP083195	Circular	1	2,599,019	2,599,019	2,371	125	99.43	0.00	37.6
HSP264	1026	Cf, I, H	2020	Spain	GCA_019997705.1	Compl	CP083194	Circular	1	2,599,017	2,599,017	2,384	155	98.86	0.00	37.6
HSP265	1026	Cf, I, H	2020	Spain	GCA_019997085.1	Compl	CP083193	Circular	1	2,599,016	2,599,016	2,369	109	99.43	0.00	37.6
HSP266	1026	Cf, I, H	2020	Spain	GCA_019997845.1	Compl	CP083192	Circular	1	2,599,010	2,599,010	2,371	204	99.43	0.00	37.6
HSP267	1026	Cf, I, H	2020	Spain	GCA_019997625.1	Compl	CP083191	Circular	1	2,598,988	2,598,988	2,374	91	99.43	0.00	37.6
HSP274	2179	Cf, P, H	2020	Spain	GCA_019998045.1	Compl	CP083190	Circular	1	2,587,513	2,587,513	2,394	230	97.82	0.00	37.6
HSP276	2179	Cf, P, H	2020	Spain	GCA_019998025.1	Compl	CP083189	Circular	1	2,587,526	2,587,526	2,386	219	98.67	0.00	37.6
HSP277	2179	Cf, P, H	2020	Spain	GCA_019997945.1	Compl	CP083188	Circular	1	2,587,535	2,587,535	2,391	316	98.30	0.00	37.6
HSP278	2179	Cf, Pa, H	2020	Spain	GCA_019997225.1	Compl	CP083187	Circular	1	2,587,540	2,587,540	2,386	196	98.30	0.00	37.6
HSP279	Unknown^*b*^	Cf, Pa, H	2020	Spain	GCA_020693965.1	Compl	CP085724	Circular^*c*^	1	2,575,504	2,575,504	2,436	139	98.56	0.00	37.8
HSP280	Unknown^*b*^	Cf, Pa, H	2020	Spain	GCA_019997045.1	Compl	CP083186	Circular^*c*^	1	2,575,827	2,575,827	2,374	137	99.41	0.00	37.8
HSP281	Unknown^*b*^	Cf, Pa, H	2020	Spain	GCA_020693985.1	Compl	CP085723	Circular	1	2,575,390	2,575,390	2,441	274	99.13	0.00	37.8
HSP282	Unknown^*b*^	Cf, Pa, H	2020	Spain	GCA_019998285.1	Compl	CP083185	Circular^*c*^	1	2,575,851	2,575,851	2,381	170	99.15	0.00	37.8
HSP283	294	Cf, I, H	2020	Spain	GCA_019997965.1	Compl	CP083184	Circular	1	2,607,096	2,607,096	2,399	154	99.43	0.00	37.6
HSP284	2180	Cf, I, H	2020	Spain	GCA_019996945.1	Compl	CP083183	Circular	1	2,568,734	2,568,734	2,361	113	99.43	1.14	37.6
HSP285	2181	Cf, Pn, H	2020	Spain	GCA_020685605.1	Contig	JAJEKG000000000	Circular	2	2,642,410	2,646,739	2,467	127	99.43	0.00	37.5
HSP286	2181	Cf, Pn, H	2020	Spain	GCA_020686685.1	Contig	JAJEKF000000000	Circular	4	2,642,415	2,650,568	2,463	144	99.43	0.00	37.5

a ID, identifier; MLST, multilocus sequence type; Cf, Canis lupus familiaris; P, perioral; Pa, perianal; I, inguinal; Pn, perinasal; H, healthy; Compl, complete genome; CDS (total), total number of coding sequences; Cov, coverage; Comp, completeness; Cont, contamination.

b MLST alleles with mutations that have not been reported.

c Genomes where the *dnaA* gene is not detected start with a random ATG gene.

A total of 51 bacterial isolates were assembled into single-circular-contig complete genomes; 16 isolates were assembled into a main circular contig corresponding to the bacterial chromosome and smaller contigs containing replicon proteins supporting their plasmid origin. Together with our previous study (3), we have announced 95 S. pseudintermedius genomes from the skin of healthy dogs, contributing to a better understanding of S. pseudintermedius pathogenesis.

### Data availability.

The standardized strain descriptions and accession numbers are presented in Table 1; the genome assemblies, genomic data, and raw data are publicly available in GenBank under BioProject PRJNA685966 and genome accession numbers CP083183 to CP083231, CP085723, CP085724, and JAJEKF000000000 to JAJEKU000000000.
